# Comparison of observed image quality and technical image quality parameters in 3D-FLAIR images

**DOI:** 10.1007/s10334-025-01292-w

**Published:** 2025-09-03

**Authors:** Juha I. Peltonen, Teemu Mäkelä, Linda Kuusela, Eero Salli, Marko Kangasniemi

**Affiliations:** HUS Diagnostic Center, Department of Radiology, University of Helsinki and Helsinki University Hospital, P.O. Box 340, FI-00029 HUS Helsinki, Finland

**Keywords:** Magnetic resonance imaging, Quality assurance, Quality control, Computer-assisted image analysis

## Abstract

**Objectives:**

Magnetic resonance imaging (MRI) is a complex medical imaging method where multiple technical and physiological factors may lead to undesired changes in image quality. The quality control methods utilizing test objects are useful in measuring technical performance, but they may not fully detect all factors present in clinical imaging. In this study, we developed methodologies to quantify observer-based image quality and to compare these observations with technical quality control (QC) parameters.

**Materials and methods:**

We analysed 150 brain MRI 3D-FLAIR volumes from 15 scanners, measuring image quality both quantitatively and by visually ranking the images using forced-choice comparison.

**Results:**

Significant differences were found between different scanners based on the forced choice comparison. In imaging study-specific analysis, a weak correlation was observed with contrast-to-noise ratio (CNR) (*R*^2^ = 0.17) and brain white matter–gray matter (WM/GM) contrast (*R*^2^ = 0.14). With device-specific median correlation, the CNR and WM/GM contrast *R*^2^ were 0.21 and 0.34, respectively. Additionally, using device-specific median values, a correlation was found with image quality index (QI) (*R*^2^ = 0.21) and some modulation transfer function (MTF) based resolution-specific parameters (MTF10 FH, *R*^2^ = 0.19; MTF10 AP, *R*^2^ = 0.20; MTF50 AP, *R*^2^ = 0.17).

**Discussion:**

The forced choice comparison can be effectively utilized to rank image quality across multiple MRI scanners. Technical image quality parameters, directly analysed from anatomical image volumes, can offer prospective maintenance value. Additionally, the quality of clinical image volumes can be assessed using both forced choice comparison and calculational image analysis methods.

**Supplementary Information:**

The online version contains supplementary material available at 10.1007/s10334-025-01292-w.

## Introduction

Magnetic resonance imaging (MRI) is a complex medical imaging technique used to visualize anatomical structures, contrasts, and biomarkers. Factors such as technical issues, user mistakes, and challenges related to patient co-operation or movement may lead to undesired changes in image appearance. A subtle decrease in image quality could lead to an unnoticed or unreported reduction in scan sensitivity.

The goal of MRI quality control (QC) is to ensure sufficient image quality. Traditionally, MRI QC has been based on the use of different test objects or phantoms. Phantoms offer static, standardized, and repeatable targets for measuring resolution, geometric accuracy, noise, signal, contrast, and other quantitative parameters. There are many commercially available QC phantoms and published studies that describe how such test objects can be constructed [1–5]. However, phantom measurements fail to represent the full complexity of MRI studies in humans. Detailed anatomical structures with varying magnetic and electrical properties, together with physiological motion, introduce image features and artefacts that are difficult to replicate in simple test objects.

These limitations can be overcome by using clinical images as a basis for QC. Image quality parameters can be obtained from patient images, e.g. by using well-defined tissues or tissue interfaces. Several authors have proposed methods for patient image-based QA: Wang et al. measured image resolution with tagged 2D images [6]. Magnotta et al. assessed signal-to-noise and contrast-to-noise ratios (CNR) in clinical 2D images [7]. Mortamet et al. followed MRI equipment performance by studying the signal in the background volume [8]. Additional methods have been presented by Osadebey et al. who based the measurements on local image entropy [9], Jang et al. with a statistical approach on image features [10], and Borri et al. who utilized the image power spectrum [11]. Hendriks et al. reviewed quality control methods for T1-weighted images [12]. Recently, Loizillon et al. presented a deep learning-based approach to classify image quality in 3D-FLAIR images [13]. Combining different metrics can be used further in quantitative performance comparisons between MRI systems [14, 15].

The relation between technical QC parameters measured from clinical images and subjective quality observed by a radiologist remains unclear. One of the challenges is that the visual characteristics defining clinical image quality are sometimes difficult to verbalize: they are often combined in the general representation of the image rather than distinct quantifiable features. Despite the vagueness, a frequently applied method for assessing image differences is to use visual scoring based on a predefined scale [16, 17]. These methodologies may fail to resolve fine differences between images due to the coarseness of the applied scales. To improve quantification, various approaches are proposed based on mutual comparison of images within a group [18–20]. Methods based on forced choice comparison are promising if the number of required ratings is high [21].

In this study, we developed methods to quantify observer-based image quality and compared these observations with QC parameters. We designed a user interface (UI) for rapid forced-choice comparisons between 3D-FLAIR head volumes. Visual assessments were used to derive image quality metrics across a cohort of MRI devices. These observer-derived metrics were then compared with the corresponding technical QC parameters obtained from the same images.

## Materials and methods

### Imaging

A three-dimensional turbo spin-echo sequence with inversion preparation, referred to as 3D-FLAIR, is an MRI sequence widely used in brain imaging protocols, e.g. for the detection of intracranial haemorrhage [22, 23]. It is generally used to detect T2 high signal findings that can be associated with various pathologic findings in the brain, especially adjacent to CSF, e.g. cortical and periventricular locations. In addition to traditional MRI sequence parameters, the flip angle of the refocusing radiofrequency (RF) pulses used to tip the magnetization can be varied throughout the echo train. Thus, the sequence’s emphasis on high-resolution targets, relaxation weighting, and CNR can be balanced [24, 25]. Due to these intricacies and differing implementations, the sequence performance regarding contrast and resolution may not be easily deductible solely from the sequence parameters available to users.

In this study we used 15 MRI scanners from three vendors. The scanners and their key technical and imaging sequence parameters are presented in Table 1. The scanners were either installed or comprehensively updated between 2005 and 2017. The field strengths were 1.5 T on 11 scanners and 3 T on four scanners. The number of elements in the used head coils varied from 8 to 64. In some scanners, there were two possible head coils with different numbers of channels. The coil selection was not controlled in these instances and likely depended on the specific study indication and patient head size. Two of the scanners, ID12 and ID15, were mobile 1.5 T units installed on trailers. None of the scanners had any known defects during the investigation period. All scanners used 3D-FLAIR sequence with constant refocusing angle or T2-relaxation—emphasizing refocusing RF pulses. The images were acquired with right–left direction as slice encoding direction, anterior–posterior as phase encoding direction and feet–head as frequency encoding direction. Parallel imaging was applied only in phase encoding direction.

**Table 1:** Scanner data and applied sequence parameters

Device	Vendor	Installation year	Field strength (T)	Head coil channels	Acquisition voxel size (mm^3^)	Typical FOV (mm^3^)	Parallel imaging factor	TE_eff_ (ms)	TR (ms)	TI (ms)	ETL	BW (Hz/pxl)
ID1	A	2017	1.5	20	1.0 × 1.0 × 1.0	250 × 219 × 192	2	335	5000	1800	214	590
ID2	A	2013	1.5	20	1.0 × 1.0 × 1.0	250 × 227 × 176	2	335	5000	1800	214	590
ID3	A	2014	1.5	20	1.0 × 1.0 × 1.0	260 × 228 × 176	2	335	5000	1800	214	590
ID4	A	2017	1.5	20	1.0 × 1.0 × 1.0	250 × 227 × 176	2	335	5000	1800	214	590
ID5	A	2016	3	32/20	1.0 × 1.0 × 1.0	250 × 250 × 176	2	394	5000	1800	256	780
ID6	A	2018	1.5	20	1.0 × 1.0 × 1.0	250 × 230 × 160	2	335	5000	1800	215	590
ID7	A	2017	3	64/20	1.0 × 1.0 × 1.0	256 × 232 × 160	2	397	5000	1800	258	780
ID8	A	2016	1.5	20	1.0 × 1.0 × 1.0	250 × 250 × 176	2	335	5000	1800	214	590
ID9	A	2011	1.5	12	1.0 × 1.0 × 1.0	250 × 242 × 176	2	335	5000	1800	266	592
ID10	A	2008	1.5	12	1.0 × 1.0 × 1.0	256 × 226 × 176	2	340	5000	1800	238	592
ID11	B	2005	1.5	8	1.0 × 1.0 × 1.2	250 × 250 × 230	2	210	5000	1660	240	943
ID12	A	2014	1.5	12	1.0 × 1.0 × 1.0	250 × 242 × 176	2	337	5000	1800	266	592
ID13	A	2014	3	20	0.9 × 0.9 × 1.2	230 × 230 × 230	2	387	5000	1800	246	750
ID14	A	2010	3	32/12	1.0 × 1.0 × 1.0	250 × 254 × 176	2	385	6000	2100	250	781
ID15	C	2009	1.5	16	1.0 × 1.0 × 1.2	256 × 256 × 208	2	140	5040	1591	260	98

*BW* receiver bandwidth, *ETL* echo train length, *TE*_eff_ effective echo time, *T* inversion time, *TR* repetition time

**Table 2:** Estimate P Shapiro–Wilk test results for each device ID

Device	*p*-value
ID1	**0.058**
ID2	0.004
ID3	0.026
ID4	0.048
ID5	**0.540**
ID6	**0.658**
ID7	**0.101**
ID8	0.032
ID9	**0.119**
ID10	**0.295**
ID11	**0.890**
ID12	**0.589**
ID13	**0.127**
ID14	**0.348**
ID15	**0.503**

The *p* values over 0.05 are bolded

### Patient population

This study included 150 retrospective 3D-FLAIR head scans from different adult patients, with ten consecutive scans taken from each of the 15 scanners. Other patient demographics or imaging indications were not controlled. All scanners used the same patient population without known bias in patient assignment. Thus, the study populations from each scanner were considered as random samples from the same overall population. This study was approved by the institutional ethics committee (HUS/2138/2019).

### Image analysis

The applied automatic QC analysis pipeline workflow was previously described by Peltonen et al. [26]. The MATLAB (The MathWorks, Natick, MA, USA) based analysis pipeline was used to extract quantitative metrics from 3D-FLAIR image volumes. Preprocessing included brain tissue segmentation and generating bias-field-corrected images. For spatial resolution assessment, the cortical surface was used as a natural boundary. The image quality parameters used in this study were modulation transfer function with 50% (MTF50) and 10% (MTF10) thresholds, white and gray matter contrast (WM/GM), CNR based on the white and gray matter contrast, noise in the brain signal, and the general image quality index (QI) based on the signal in the background area of the imaging volume [8].

The following small improvements to the previously presented analysis pipeline were applied to enhance compatibility with different scanner field strengths, vendors, and image properties. The inclusion of the 1.5-T scanners required an adjusted brain segmentation template. Minor vendor-specific differences in DICOM metadata had to be matched. Also, due to slight differences in contrasts, the resolution calculation was adapted to the base signal levels.

### Observed image quality

**Fig. 1:**
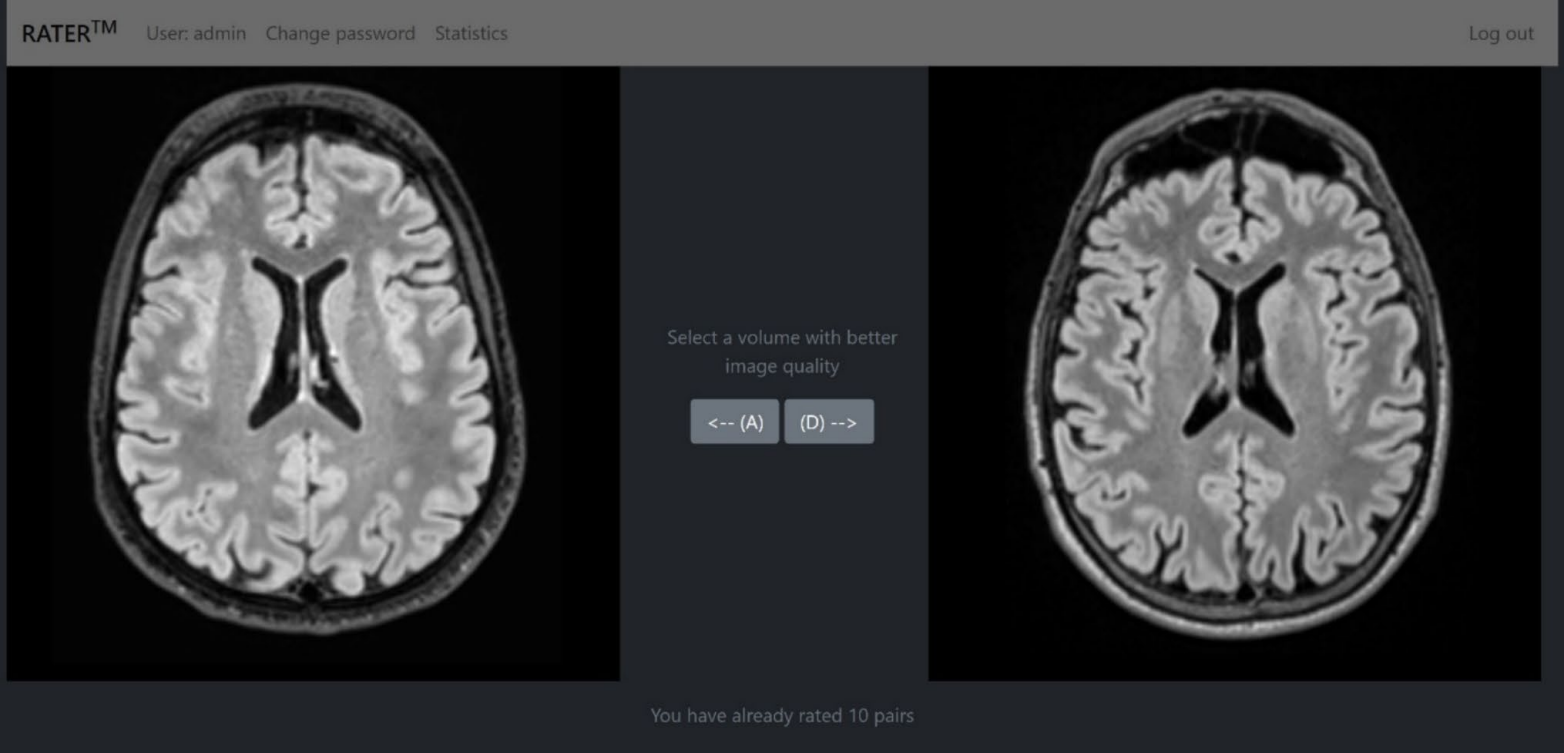
The user interface for image quality forced choice

The visually observed image quality was quantified using a blinded forced choice on a pair of image volumes. A dedicated web-based UI was built to enable rapid comparison between images. The interface used Django [27] as a backend to handle database and web-page template operations. The frontend was built using Bootstrap [28] for layout and Cornerstone [29] for DICOM image presentation. The UI offered the user a view of two scrollable transversal 3D-FLAIR image stacks. The user had to choose which of the volumes, left or right, had better image quality. After the selection, the vote was recorded to a PostgreSQL [30] database and a next random pair of volumes was loaded on the screen. The only rule limiting the randomization was that the volume could not be compared to itself. A screenshot of the UI frontend is shown in Fig. 1.

The blinded forced choice evaluation was performed by one senior neuroradiologist with over 20 years of experience in neuroradiology (MK). The total number of forced-choice comparisons was 2000.

### Clinical image quality calculation

The clinical quality of each brain volume was defined as estimate P presented by Burgess [21]. The estimate P for a volume being graded superior to another volume in the same sample group was calculated by equation:

1 $$P = \frac{{N_{s} }}{{N_{t} }},$$

where N_s_ is the number of comparisons with the volume being voted superior and N_t_ is the total number on comparisons with the volume. Consequently, estimate P follows a binominal distribution and the standard deviation (SD) can be calculated by equation:

2 $$SD = \sqrt {\frac{{P\left( {1 - P} \right)}}{{N_{t} }}} .$$

The estimate for observed clinical quality for each volume was calculated by Eq. 1 with standard deviation according to Eq. 2.

### Statistical tests

Statistical analysis was performed using MATLAB (The MathWorks Inc, Natick, Massachusetts). A Shapiro–Wilk test (*p* > 0.05) was used to test the normality of the estimate P sample normality corresponding to each device. A single-factor analysis of variance (ANOVA) was used to test statistical differences in estimate P grouped according to MRI scanner. Tukey’s honestly significant difference (HSD) test was applied to compare each scanner with the others while adjusting for multiple comparisons. A *p*-value ≤ 0.05 was considered statistically significant.

The coefficient of determination (*R*^2^) was calculated between the observed image quality and each technical parameter based on linear correlation. The *R*^2^ was calculated based on each study as well as based on the median values of each device.

## Results

**Fig. 2:**
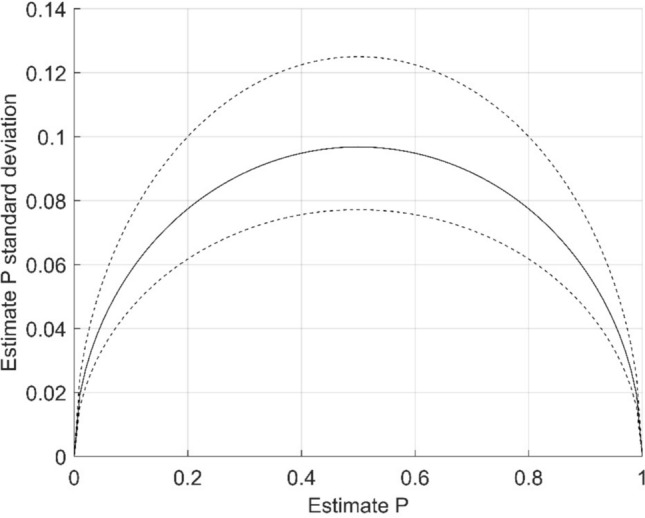
Estimate P standard deviation. Upper and lower limit of the standard deviation presented as dashed line and mean of the standard deviation as continuous line

The total number of forced-choice comparisons was 2000: 235–293 (mean 266.7) per scanner and 16–42 (mean 26.7) per study. Based on Eq. 2, the standard deviation of the study estimate P was typically under 0.1 and in all cases under 0.13. The range of standard deviations of a single study P estimate is presented in Fig. 2.

**Fig. 3:**
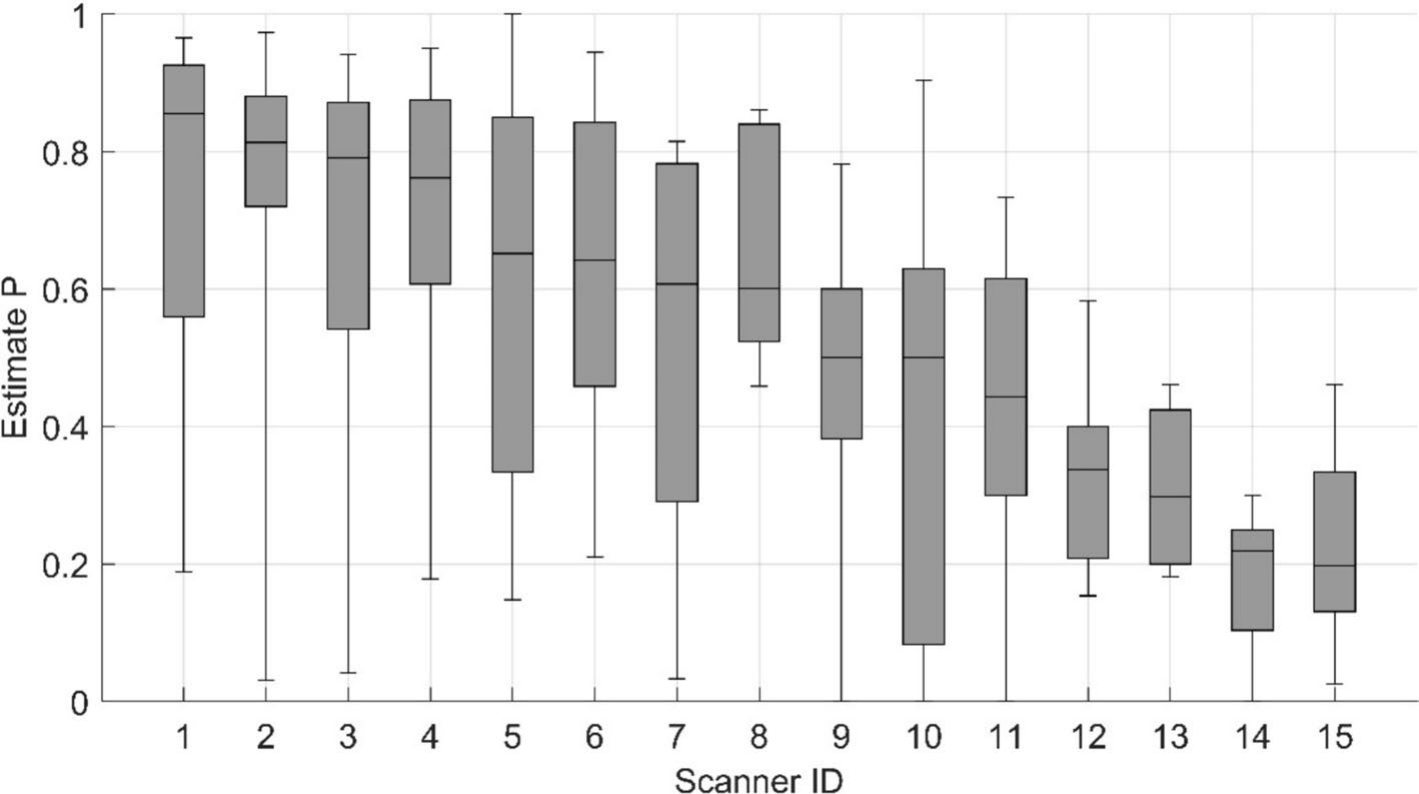
Device-specific estimates P boxplot. The median of the P is presented as a horizontal line, the 25th and 75th percentile of the P is presented as the lower and upper limits of the box, respectively, and the whiskers represent minimum and maximum values

The device-specific estimate *P* values are presented in Fig. 3. Table 2 presents the Shapiro-Wilk test results for the image quality estimate P across different device IDs.The ANOVA revealed a significant difference in device-specific estimate *P* values (*p* < 0.05). The pairwise statistical differences identified by Tukey’s HSD test are presented in Supplemental Table 1.

When devices are arranged in descending order based on the median estimate P, those ranked highest display a statistically significant difference in estimate P compared to those ranked lowest (*p* < 0.05). The difference in the observed image quality was not statistically significant in between any closely ranked device.

**Fig. 4:**
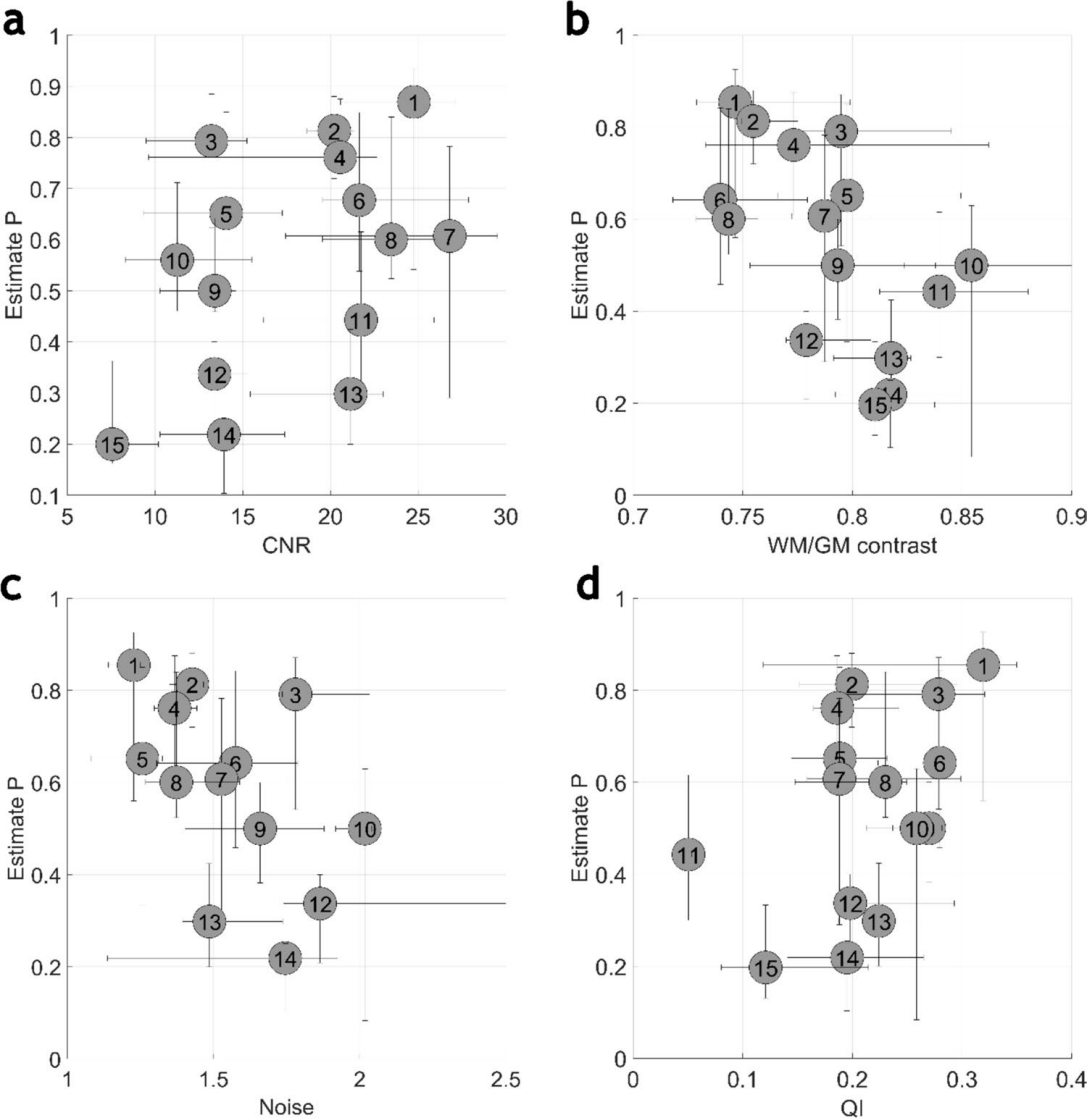
The device-specific plot of CNR (**a**), contrast (**b**), noise (**c**) and QI (**d**) parameters and estimates P. A circle with respective device ID is representing the median value of the P estimate and technical image quality parameters. The horizontal and vertical whiskers attached to each circle are presenting the 25th and 75th percentile of the values

**Fig. 5:**
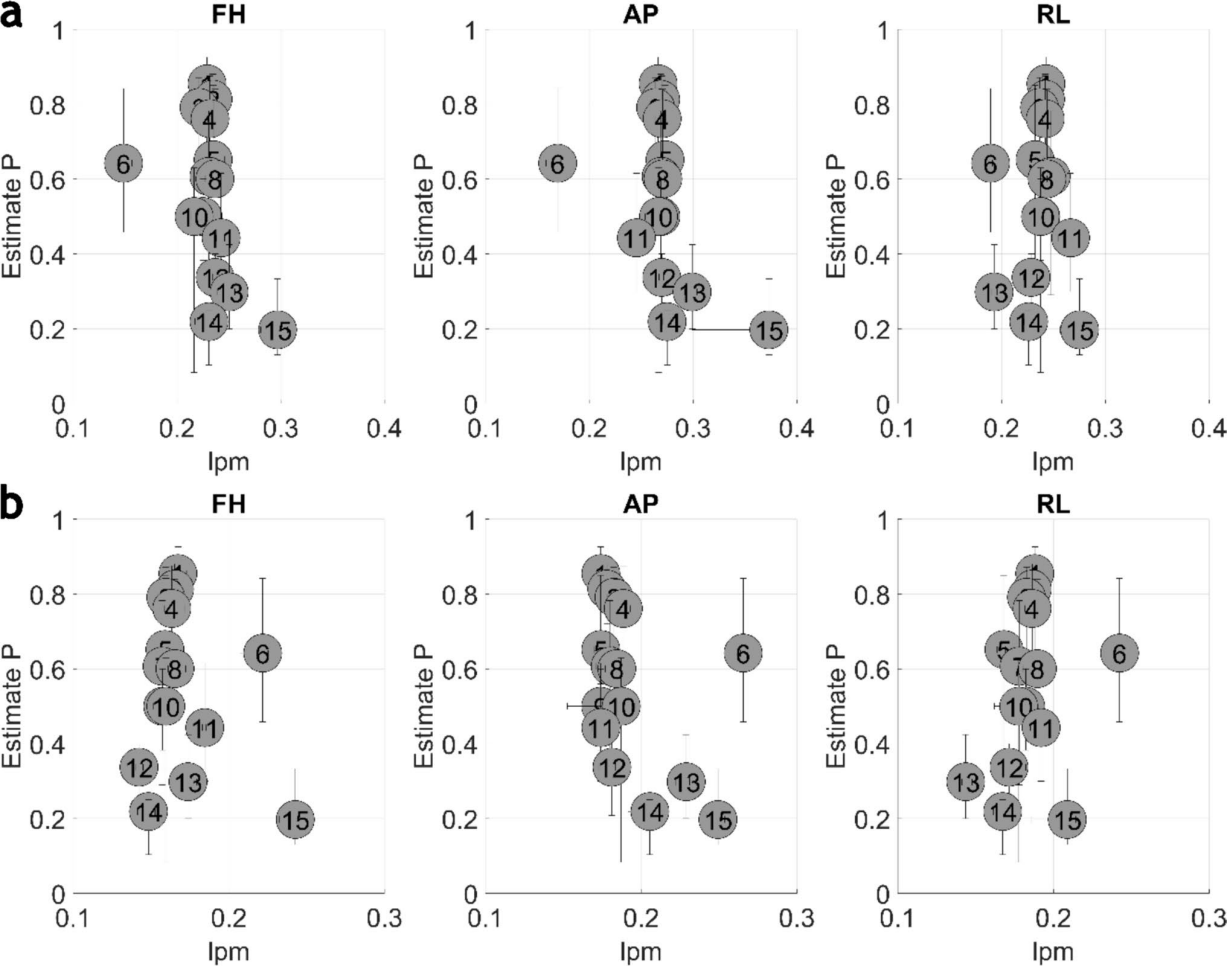
The device-specific plot of MTF10 (**a**) and MTF50 (**b**) parameters and estimates P. A circle with respective device ID represents the median value of the P estimate and technical image quality parameters. The horizontal and vertical whiskers attached to each circle are presenting the 25th and 75th percentile of the values

The device-specific technical image quality parameters and estimate P values are presented in Fig. 4 and 5. Generally, MRI devices with better estimate P also achieve better values with technical QC parameters.

The *R*^2^ of study-specific technical image quality parameters and estimate *P* were limited in all cases (*R*^2^ < 0.18) as well as with all device-specific median values (*R*^2^ < 0.34). In study-specific correlation, weak correlation was observed with CNR (*R*^2^ = 0.17) and WM/GM contrast (*R*^2^ = 0.14). With the device-specific median correlation, the CNR and WM/GM contrast *R*^2^ were 0.21 and 0.34, respectively. Additionally, with device-specific median values, correlation was found with QI (*R*^2^ = 0.21) and some of the resolution-specific parameter (MTF10 FH, *R*^2^ = 0.19; MTF10 AP, *R*^2^ = 0.20; MTF50 AP, *R*^2^ = 0.17). The full coefficient of determination metrics is presented in Table 3.

**Table 3:** The coefficient of determination between each QC parameter on P estimates by study and by device-specific median values

QC parameter	*R*^2^ by study	*R*^2^ by device median
QI	0.00	0.21
Contrast	0.14	0.34
Noise	0.00	0.02
CNR	0.17	0.21
MTF10 FH	0.03	0.19
MTF10 AP	0.02	0.20
MTF10 RL	0.01	0.00
MTF50 FH	0.00	0.05
MTF50 AP	0.00	0.17
MTF50 RL	0.08	0.05

## Discussion

In this study, we have presented a methodology to compare the observed image quality of MRI image volumes and technical image QC parameters derived from the same volumes. We applied the forced choice method to quantify observed image quality and to differentiate devices from each other. A limited correlation with observed image quality and technical parameters was shown, both by study and by device.

The complex relation between medical image quality and technical QC parameters is difficult to quantify, where the key challenge is in converting the ambiguous differences in observed image quality to measurable parameters. With the presented methodology, based on blinded forced choice, we were able to show significant differences in the observed image quality between the devices operating with similar MRI sequence acquisition parameters.

The grading method, based on a predetermined scale, requires experienced and calibrated observers and eventually offers only limited specificity. Meanwhile, these problems can be partly overcome by the forced choice method. By increasing the number of votes, the accuracy of the estimate *P* values can be increased and the uncertainty of the image quality estimate decreased. In the present study, we used a single experienced observer, but the method could accommodate multiple observers. The bias between observers can be statistically controlled as part of the analysis [31]. Increasing the number of observers and votes can significantly scale up the survey, allowing it to reach study population sizes previously unfeasible with a predetermined scale-based grading.

As the power of the method lies in the number of votes; however, dedicated UI should be used to reach a maximum amount of data with a reasonable workload. With the presented UI, an experienced observer can provide a vote on a simplified question about the image volume within seconds, making the total yield of a few thousand votes reachable even with a single observer. The voting platform can be further improved to increase usability and engagement, even by including gamification elements.

The applied methods to measure the technical image quality of the image volumes have been shown to respond to changes in the clinical images [26]. A limited *R*^2^ with the observed image quality and technical image quality parameters was found in this study. While devices with better technical image quality also received a better estimated observed image quality, this correlation was not guaranteed. Compared with device-specific median values, the *R*^2^ in study-specific comparison was weaker. Study-specific *R*^2^ in contrast-based QC metrics was generally higher than in MTF-based metrics, indicating the higher role of contrast in the observed image quality with image volumes.

The QC parameter related only to noise, lacking correlation with the observed image quality, implying the level of noise is within an acceptable limit for image interpretation. The relationship between image quality and noise may not be linear but appears to have a threshold of effect.

The weaker study-specific correlation compared with the device-specific correlation is likely due to substantial study-by-study variation. Especially, nuisance features contributing to MTF-specific technical QC parameters may originate either from the device’s technical performance or patient motion artefacts. However, the same feature may have a fundamentally different impact on the observed image quality. The motion artefact can be perceived as a natural feature of an image while the reduced resolution due to technical performance may appear unnatural.

A better ranking in observed image quality was generally associated with the more recently installed devices, based on blinded quantification. This is an interesting finding supporting the effect of technological advancement. To be more specific, scanners with ID1–ID8 had improved RF system with a higher number of coil channels and signal digitization closer to the patient whereas scanners with ID9–ID15, except ID14, had not. Scanner ID14 had an advanced RF system, but the acquisition voxel size deviated from the dominant setting. The digitalization of the MRI scanner’s RF system has been shown before to improve the CNR of the brain images [32].

The results show that the observed image quality of the devices varied significantly, even with almost identical scan protocol parameters. Higher main magnetic field strength is normally seen as a way to increase signal and contrast, especially in brain imaging. However, it did not guarantee better observed image quality in this study. Theoretically, a higher field strength should allow an increase in spatial resolution while maintaining acceptable CNR and consequently increase the observed image quality. The ETL affects the high image frequency components, contributing edges to the image, and also the image contrast due to increased T2 weighting and decreased signal strength of later echoes in the train. In general, devices with lower ETL obtained better ranking in observed image quality. In our study, three devices (ID11, ID14 and ID 15) showed a considerable variation from the typical settings in effective echo time, repetition time (TR), or inversion time (TI). With the device IDs 11 and 15, this variation was likely due to vendor-specific sequence design and default settings. The reason behind TR and TI variation with the device ID14 remains unknown. In all three cases, deviating sequence parameters may have contributed to lower rankings in observed image quality.

The accuracy of the observed image quality analysis is limited by the number of votes per study and patient-by-patient variation in the sample data. In theory, the required number of votes can be determined by the planned maximum allowed variation in the image quality estimate. On the other hand, the variation in values can be calculated directly from the final image quality estimates. However, the relation between statistical variation and patient-induced variation is difficult to determine, especially if both are estimated to be in the same range. The effect of statistical variation can be decreased by increasing the number of votes. The patient-by-patient variation may be reduced through a more educated choice of patient population. In this study, the patients on each scanner were considered a random sample from the same population. There was no control on demographic parameters other than ensuring all patients were adults and thus portraying a relevant clinical setting. The impact of demographic factors on the image quality offers an interesting topic for further research requiring a significantly larger study population than presented in this study.

The statistics in forced choice experiments follow binomial distribution which approaches normal distribution with a sufficiently large sample size. With Shapiro–Wilk test, image quality estimate P was shown to be normally distributed with all but four devices (IDs 2, 4, and 8). While the reason for this deviation is unclear, it may be related to patient-by-patient variation. For example, contrast decreasing motion artefact could induce reduction in image quality deforming the distribution.

## Conclusions

Forced choice can be used to quantify observed image quality accurately. The connection between observed image quality and clinical image derived technical image quality was proven, but offers only limited correlation.

## Supplementary Information

Below is the link to the electronic supplementary material.

Supplementary Supplemental Table 1. The device specific pairwise statistical differences of estimate P identified by Tukey’s HSD test. Values under 0.05 are bolded. file1 (XLSX 19 KB)

## Data Availability

The datasets analysed during the current study are not publicly available due to privacy concerns. The software for interactive forced-choice comparisons is available from the corresponding author upon a reasonable request.
